# Predictors of Treatment Failure among Adult Antiretroviral Treatment (ART) Clients in Bale Zone Hospitals, South Eastern Ethiopia

**DOI:** 10.1371/journal.pone.0164299

**Published:** 2016-10-07

**Authors:** Demewoz Haile, Abulie Takele, Ketema Gashaw, Habtamu Demelash, Dabere Nigatu

**Affiliations:** 1 School of Public Health, College of Health Sciences, Addis Ababa University, Addis Ababa, Ethiopia; 2 Department of Nursing, College of Medicine and Health Sciences, Madda Walabu University, Bale Goba, Ethiopia; 3 Department of Public Health, College of Medicine and Health Sciences, Debre Tabor University, Debre Tabor, Ethiopia; Temple University School of Medicine, UNITED STATES

## Abstract

**Background:**

Treatment failure defined as progression of disease after initiation of ART or when the anti-HIV medications can’t control the infection. One of the major concerns over the rapid scaling up of ART is the emergence and transmission of HIV drug resistant strains at the population level due to treatment failure. This could lead to the failure of basic ART programs. Thus this study aimed to investigate the predictors of treatment failure among adult ART clients in Bale Zone Hospitals, South east Ethiopia.

**Methods:**

Retrospective cohort study was employed in four hospitals of Bale zone named Goba, Robe, Ginir and Delomena. A total of 4,809 adult ART clients were included in the analysis from these four hospitals. Adherence was measured by pill count method. The Kaplan Meier (KM) curve was used to describe the survival time of ART patients without treatment failure. Bivariate and multivariable Cox proportional hazards regression models were used for identifying associated factors of treatment failure.

**Result:**

The incidence rate of treatment failure was found 9.38 (95% CI 7.79–11.30) per 1000 person years. Male ART clients were more likely to experience treatment failure as compared to females [AHR = 4.49; 95% CI: (2.61–7.73)].Similarly, lower CD4 count (<100 m^3^/dl) at initiation of ART was found significantly associated with higher odds of treatment failure [AHR = 3.79; 95% CI: (2.46–5.84).Bedridden [AHR = 5.02; 95% CI: (1.98–12.73)] and ambulatory [AHR = 2.12; 95% CI: (1.08–4.07)] patients were more likely to experience treatment failure as compared to patients with working functional status. TB co-infected clients had also higher odds to experience treatment failure [AHR = 3.06; 95% CI: (1.72–5.44)]. Those patients who had developed TB after ART initiation had higher odds to experience treatment failure as compared to their counter parts [AHR = 4.35; 95% CI: (1.99–9.54]. Having other opportunistic infection during ART initiation was also associated with higher odds of experiencing treatment failure [AHR = 7.0, 95% CI: (3.19–15.37)]. Similarly having fair [AHR = 4.99 95% CI: (1.90–13.13)] and poor drug adherence [AHR = 2.56; 95% CI: (1.12–5.86)]were significantly associated with higher odds of treatment failure as compared to clients with good adherence.

**Conclusion:**

The rate of treatment failure in Bale zone hospitals needs attention. Prevention and control of TB and other opportunistic infections, promotion of ART initiation at higher CD4 level, and better functional status, improving drug adherence are important interventions to reduce treatment failure among ART clients in Southeastern Ethiopia.

## Introduction

Treatment failure can be defined as progression of disease after initiation of ART. Treatment failure happens when the anti-HIV medications can’t control the infection. Treatment failure might happen in the form of: virologic failure, immunologic failure, and clinical progression either in combination or discordantly [1]. People with HIV run a higher risk of virologic failure than previously thought, even when their number of Ribose Nucleic Acid (RNA) copies of the retrovirus per milliliter of blood is slightly above the detection threshold [2]. A 2012 WHO estimate showed that possible HIV drug resistance in African region is above 18%.Pooled estimates from eight African countries showed that the prevalence of HIV drug resistance among people experiencing first-line therapy failure at a median duration of 12 months was 62%(95% CI: 47–77) [3].

Treatment failure is associated with high risk of mortality among ART clients [4–6]. Patients who developed HIV drug resistance in the first year of treatment had higher risk of mortality [4]. Many studies showed evidence for an increased risk of progression to AIDS or death among those with poor immune recovery [7–9].

In resource poor setting like Ethiopia, ART treatment is not guided by viral load and genotypic testing at the start of treatment rather only by CD4 count and clinical progression. There are few studies in Ethiopia regarding the rate of treatment failure. A study conducted at Debremarkos Hospital, Northwest Ethiopia revealed that 21% of patients had developed immunological failure with a failure rate of 8 per 100 patient-years of follow up [10]. Despite the few studies conducted in Ethiopia, the predictors of treatment failure among adult ART users are not well explored. Moreover the predictors of the treatment failure were not consistent across studies. Thus, this study aimed to identify the predictors of treatment failure among ART clients attending at Bale zone Hospitals. Identifying rate of treatment failure and associated factors would assist clinicians to provide targeted clinical and immune-based treatment approach. Additionally, this study would assist the effort to reduce high mortality among ART clients at the national level by improving the treatment success.

## Methods

### Ethical consideration

Ethical clearance was obtained from the Institutional Research Ethics Review Committee (IRERC) under the research and community service directorate office of Madda Walabu University. A letter of permission was obtained from zonal health department and finally willingness from each hospital was obtained to access the ART clients’ database. Only card number was used to identify cards and information collected from clients’ cards were kept anonymous and confidential.

### Study setting, design and sampling procedure

**Fig 1:**
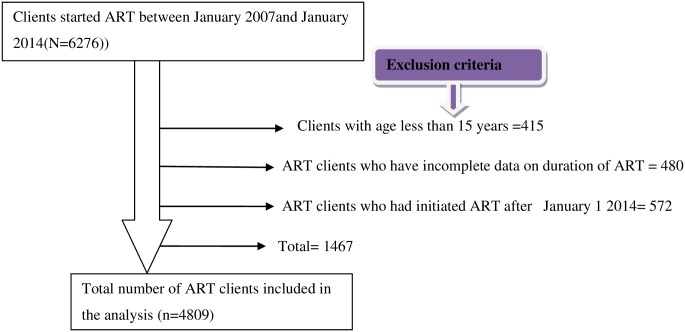
Diagram showing the final sample size included in the study between January 2007 and January 2014.

Retrospective cohort study design was employed in four hospitals of Bale zone, Southeastern Ethiopia, named Goba hospital, Ginir hospital, Robe hospital and Delomena hospital. Goba hospital was the first hospital in Bale zone to initiate ART service at 2005/6. The ART service has further expanded later to other three hospitals such as Delomena, Ginir and Robe hospitals. Currently, there are 3197 ART clients in Goba hospital, 1381 ART clients in Robe hospital while there are 234 and 1464 clients in Delomena and Ginir hospitals, respectively. Totally 6276 HIV patients had been enrolled in the ART program [11]. This study was conducted on Adult (≥15 years old) ART users. The total numbers of ART clients included in this study were 4809.Alladult ART clients who started treatment on these seven years between January 1, 2007 and December 31, 2014 were included in the study. The medical records of adult HIV patients (≥15 years) who initiated ART between 2007 and 2014 were included in the study. The schematic presentation of the sampling procedure is presented in Fig 1.

### Data collection tool and quality control procedures

The federal ministry of health (FMOH) ART follow up form was used as a data extraction format. The data were extracted by the data clerk working at each hospital who took training for two days. Data extraction process was supervised by the principal investigators and trained supervisors. Data check was done on the collected data randomly by extracting the data of 2% patients from each hospital data base and compare with collected data by the data clerks. Totally, 368 ART clients’ data were checked against the data with the ART data base system by the principal investigators. The collected data were checked for completeness prior to data entry. Finally, data exploration on entered data was made to see unexpected values, outliers, and identify variables which need transformation.

### Variables extracted and definitions

Socio-demographic characteristics (age, sex, educational status, marital status), duration on ART (measured in months), anthropometric measurements (weight in Kg and height in meter), WHO clinical staging (I-IV), CD4 count, functional status, presence of opportunistic infection including TB, drug regimen, presence of drug substitute, regimen change, regimen stopped presence of reported side effect and drug adherence were extracted from the data base.

Treatment duration for this study is the time of follow-up elapsed after ART initiation until the patient develops outcome of interest (treatment failure which was measured in months). The definition of each clinical stage was based on the WHO classification for AIDS clients according to AIDS related sign and symptoms the patient experiencing as stated in the national guideline [12]. Based on the Ethiopian ART guideline if an ART client had not seen for ≥ 1 month, the client considered as lost. However, if a client had not seen for ≥ 3 months, he/she would be recorded as a dropout. A client who is transferred to another health facility for care was recorded in the database system as transferred out. For this study, any ART client who was recorded as lost to follow-up, transferred out or dropped-out was considered as censored. The functional status was categorized based the definition stated on the ART follow up card. Working functional status clients were clients who are able to perform usual work in or out of the house while ambulatory functional status clients refer to clients who able to perform activities of daily living. Bedridden functional status was designate to clients who are not able to perform activities of daily living. Adherence was measured based on the pills count when she /he comes for the follow up and recorded on the follow up forms either good, fair or poor. A patient was categorized as having good adherence if pill count or self reported adherence was greater than or equal to 95%. Fair adherence: if pill count or self reported adherence was less than 85%-95% while poor adherence: if pill count or self reported adherence was less than <85%.

### Outcome measure

The treatment failure was classified retrospectively from what was reported by clinicians in patient’s clinical charts. A client was categorized treatment failure by a clinician as if he or she met one of the following three criteria after at least six months of follow up (1) a fall of CD4 count to pre-therapy baseline or below or 50% fall of absolute CD4 count from the on-treatment peak value (2) persistent CD4 levels below 100 cells/mm^3^ (3)the occurrence or recurrence of HIV-related events after at least 3 months of treatment (clinical disease progression with development of an opportunistic infection) with the exception of immune reconstitution syndromes.

### Statistical analysis

The statistical analysis was done by STATA version 12. Descriptive statistics such as frequency, median, inter-quartile range (IQR), mean and standard deviation (±SD) were computed for all continuous and categorical variables. Person time (years) contribution of each study participant were calculated by comparing duration on ART and treatment failure as an outcome variable.

The Kaplan Meier (KM) curve with log rank test was used to describe the probability of survival without treatment failure. In order to identify the predictors of treatment failure, bivariate and multivariable Cox proportional hazards regression models were employed. The variables we chose for regression model as predictors were extracted from the ART data base. The reported predictors of treatment failure from literatures were not exactly the same, different literatures reported different predictors of treatment failure. Thus we have tested all the potential predictor variables extracted from the ART data base system in the regression.

Those statistically significant variables in bivariate analysis at p-value <0.25 [13] were entered into multivariable Cox proportional hazard regression model to identify the independent predictors of treatment failure. Both crude and adjusted hazard ratios (HRs) with 95% confidence intervals were reported and variables with p-values<0.05 in the multivariable Cox regression model were considered statistically significant factors of treatment failure.

## Results

### Characteristics of ART clients with treatment failure and success

This study included 4,809 adult ART clients from the four hospitals of Bale Zone. Majority of (57.64%) ART clients had attended their follow up at Goba Hospital. The median (IQR) age at ART initiation among adults cohorts was 33 years (IQR = 28–40). The highest proportion of treatment failure is found among ART clients attending in Delomena Hospital (6.47%) and among ART clients with age 45 years and above (5.24%). The prevalence of treatment failure among male ART clients was 4.37% while the prevalence of treatment failure was 0.72% among female ART clients. From ART clients with primary education level, the prevalence of treatment failure was found to be 2.91% while it was 2.74% among ART clients who had divorced from their partner (Table 1).

**Table 1:** Socio-demographic characteristics of HIV patients who had ART treatment failures and success in Bale Zone hospitals, South East Ethiopia; 2015.

Variables	Treatment failure		Treatment successful	
	Number	Percent	Number	Percent
**Hospitals**				
Goba	71	2.56	2,701	97.44
Robe	6	0.59	1,003	99.41
Delomena	9	6.47	130	93.53
Ginnir	27	3.04	862	96.96
**Age (years)**				
15–24	0	0	606	100
25–34	31	1.60	1,912	98.40
35–44	37	2.64	1,365	97.36
≥ 45	45	5.24	813	94.76
**Sex of participants**				
Male	94	4.37	2,059	95.63
Female	19	0.72	2,637	99.28
**Educational level**				
No Education	13	1.17	1,100	98.83
Primary	54	2.91	1,800	97.09
Secondary and above	46	2.72	1,648	97.28
**Marital status**				
Never married	14	2.41	566	97.59
Married	75	2.50	2,931	97.50
Divorced	19	2.74	675	97.26
Widowed	5	1.23	403	98.77

The descriptive statistics showed that about 4% of ART clients who had bedridden functional status at ART initiation experienced treatment failure while 2.29% ART clients who had ambulatory functional status at ART initiation experienced treatment failure.

None of ART clients with WHO clinical stage I at ART initiation experienced treatment failure. In contrary about 7% of ART clients with WHO clinical stage IV at ART initiation experienced treatment failure. The prevalence of treatment failure among ART clients who initiated ART at CD4 ≤200 per millimeter cube of blood was 2.51%. A total of 2.48% ART clients who had initiated ART at CD4 ≥300 per millimeter cube of blood experienced treatment failure. Among those who had TB—confection at ART initiation, nearly 4% had ART treatment failure while among ART clients who had developed TB after ART initiation, 7.77% of them had experienced treatment failure. Above 11% of ART clients with opportunistic infection at ART initiation had treatment failure. The proportion of ART clients with treatment failure among those who had normal BMI category were 3.17% (Table 2).

**Table 2:** Clinical characteristics of HIV patients who had ART treatment failures and success in Bale Zone hospitals, South East Ethiopia; 2015.

Variables	Treatment failure		Treatment successful	
	Number	Percent	Number	Percent
Baseline* functional status				
Working	85	2.25	3,686	97.75
Ambulatory	18	2.29	768	97.71
Bedridden	10	4.02	239	95.98
**WHO stage at baseline**				
WHO Stage 1	0	0	683	100
WHO stage 2	16	1.82	865	98.18
WHO stage 3	70	2.44	2,800	97.56
WHO stage 4	27	7.20	348	92.80
**Initial CD4 Count**				
≤200	72	2.51	2,800	97.49
201–300	14	1.61	854	98.39
>300	16	2.48	628	97.52
**TB co-infection at initiation of ART**				
Positive	21	3.95	510	96.05
Negative	92	2.15	4,186	97.85
**Patient develop TB after ART initiation**				
Yes	8	7.77	95	92.23
No	105	2.23	4,601	97.77
**Presence of other opportunistic infection**				
Yes	9	11.54	69	88.46
No	104	2.20	4,627	97.80
**BMI at Initial visit**				
<18.5	36	1.81	1,958	98.19
18.5–24.9	76	3.17	2,321	96.83
≥25.0	1	0.33	302	99.67

A total of 16.36% of ART clients with fair drug adherence at the first visit after ART initiation experienced treatment failure. However among those who had poor adherence at the first visit after ART initiation, below 2% of ART clients experienced treatment failure.

Among those ART clients who had drug regimen change experience, the prevalence of treatment failure was found 6.63% while the prevalence of treatment failure among those ART clients who had an experience of drug substitute was 4.17%. The highest prevalence of treatment failure was observed among ART clients who used 1d (AZT+3TC+EFV) (8.43%) regimen while the lowest prevalence of treatment failure was 1.53% among ART clients who were on 1e (TDF+3TC+EFV)drug regimen. The prevalence of treatment failure among those ART clients who had reported drug side effect was found 2.88%. A total of 16.22% of treatment failure was occurred among ART clients who had reported fatigue as a drug side effect (Table 3).

**Table 3:** Drug related characteristics of HIV patients who had ART treatment failures and success in Bale Zone hospitals, South East Ethiopia; 2015.

Variables	Treatment failure		Treatment successful	
	Number	Percent	Number	Percent
**Drug adherence at the first visit after ART initiation**				
Good	89	2.18	3,992	97.82
Fair	9	16.36	46	83.64
Poor	7	1.35	510	98.65
**Is there drug regimen change**				
Yes	108	6.63	1,522	93.37
No	5	0.16	3,174	99.84
**First line ARV drug regimen**				
1a(d4T+3TC+NVP)	49	3.10	1,531	96.90
1b(d4T+3TC+EFV)	12	3.19	364	96.81
1c(AZT+3TC+NVP)	19	1.64	1,138	98.36
1d(AZT+3TC+EFV)	7	8.43	76	91.57
1e(TDF+3TC+EFV)	15	1.53	963	98.47
1f(TDF+3TC+NVP)	11	1.78	607	98.22
**Drug substitute**				
Yes	67	4.17	1,538	95.83
No	43	1.35	3,154	98.65
**Presence of reported side effect**				
Yes	6	2.88	202	97.12
No	107	2.33	4,494	97.67
**Reported side effects**				
Fatigue	6	16.22	31	83.78
Others*	0	0	168	100
**Regimen stopped**				
Yes	4	80.00	1	20.00
No	108	2.25	4,695	97.75

*Headache, nausea, numbness, rash, Anemia, Abdominal pain, Dizzy, anxiety, nightmare and depression.

### Treatment failure

**Fig 2:**
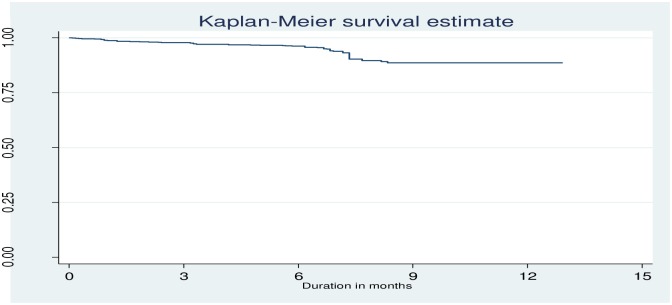
Survival of ART clients without treatment failure among a cohort of ART clients; Bale zone hospitals, Southeast Ethiopia; 2015.

In this ART cohort, there were 113treatment failures in11,830 person years of retrospective follow up. This makes the incidence rate of treatment failure 9.38 (95% CI: 7.79–11.30) per 1000 person years. As shown in Fig 2, the treatment failure occurred before 10 months of duration on ART. The highest rate of treatment failure was occurred between 6 months and 10 months of ART initiation (Fig 2).

### Predictors of treatment failure

The multivariable Cox proportional hazards regression analysis showed that older age clients, male ART clients, bedridden clients, ambulatory clients, TB co-infected clients at ART initiation, clients who develop TB co-infection after ART initiation, type of drug regimen used, drug adherence, clients with low CD4 count, and clients who had developed other opportunistic infection had a statistically significant association with treatment failure among ART clients. Male ART clients were 4.49 times more likely to experience treatment failure as compared to females ART clients [AHR = 4.49; 95% CI: (2.61–7.73)]. The multivariable model showed the estimated risk of treatment failure increases 2.91 times if the ART client is a year older [AHR = 2.91; 95% CI: (2.82–2.97)]. Similarly, lower CD4 count at initiation of ART was found significantly associated with higher odds of treatment failure [AHR = 3.79; 95% CI: (2.46–5.84)].Bedridden patients were 5.02 times more likely to experience treatment failure as compared to patients with working functional status [AHR = 5.02; 95% CI: (1.98–12.73)]. Those ART clients with ambulatory functional status were 2.12 times more likely to experience treatment failure as compared to clients with working functional status [AHR = 2.12; 95% CI: (1.08–4.07)]. TB co-infected clients were 3.06 times more likely to experience treatment failure [AHR = 3.06; 95% CI: (1.72–5.44)]. Those ART clients who had developed TB after ART initiation had higher odds to experience treatment failure as compared to their counterparts [AHR = 4.35; 95% CI: (1.99–9.54)].

ART clients who had other opportunistic infection during ART initiation were 7 times more likely to experience treatment failure [AHR = 7.0; 95% CI: (3.19–15.37)]. Similarly having fair drug adherence was significantly associated with higher odds of treatment failure [AHR = 4.99 95% CI: (1.90–13.13)]. ART clients with poor drug adherence had higher odds to experience treatment failure as compared to clients with good adherence [AHR = 2.56; 95% CI: (1.12–5.86)]. ART clients who had used regimen 1b(d4T+3TC+EFV) [AHR = 0.29; 95% CI:(0.12–0.69)]and 1c(AZT+3TC+NVP) [AHR = 0.45; 95% CI: (0.22–0.89)] had lower odds of experiencing treatment failure as compared to clients who had used regimen 1a(d4T+3TC+NVP). However, ART clients who had used regimen 1d(AZT+3TC+EFV)[AHR = 4.47; 95% CI: (1.81–11.04)] had higher odds of experiencing treatment failure as compared to clients who had used regimen 1a (d4T+3TC+NVP) (Table 4).

**Table 4:** Bivariate and multivariable cox regression model on factors associated with treatment failure among ART clients in South-eastern Ethiopia, 2015.

Variables	Treatment failure as an outcome	
	@Unadjusted Hazard Ratio (95% CI)	*Adjusted Hazard Ratio (95% CI)
**Age of the ART client**	2.89(2.86–2.92)	2.91(2.82–2.97)
**Sex**		
Male	1.21 (1.14–1.28)	4.49 (2.61–7.73)
Female	Reference	Reference
**Marital status**		
Never married	Reference	Reference
Married	0.94(0.86–1.03)	0.97(0.37–2.48)
Widowed	0.89(0.79–0.99)	1.88(0.67–5.30)
Divorced	0.88(0.78–1.00)	1.41(0.29–6.99)
**Functional status**		
Working	Reference	Reference
Ambulatory	1.0(0.92–1.08)	2.12 (1.08–4.07)
Bedridden	1.97(1.03–3.81)	5.02 (1.98–12.73)
**Baseline WHO stage**		
WHO Stage 1	Reference	Reference
WHO Stage 2	0.89(0.37–2.16)	0.91(0.82–1.02)
WHO stage 3	1.38 (0.674–2.83)	0.95 (0.86–1.04)
WHO stage 4	4.11(1.82–9.31)	0.86(0.75–1.003)
**CD4 Count at ART initiation**		
< 100 m^3^/dl	3.08(2.12–4.47)	3.79 (2.46–5.84)
≥100 m^3^/dl	Reference	Reference
**TB co-infection**		
Positive	2.42(1.51–3.89)	3.06 (1.72–5.44)
Negative	Reference	Reference
**Developing TB after ART initiation**		
Yes	3.86(1.87–7.95)	4.35(1.99–9.54)
No	Reference	Reference
**Other opportunistic infection at ART initiation**		
Yes	7.53(3.81–14.90)	7.0(3.19–15.37)
No	Reference	Reference
**Baseline BMI (kg/m**^**2**^**)**		
<18.5	1.72 (1.161–2.57)	1.5(0.95–2.35)
18.5–24.99	Reference	Reference
≥25.0	0.201 (0.03–1.47)	0.18(0.03–1.37)
**Adherence at first visit after ART initiation**		
Good	Reference	Reference
Fair	7.52(3.77–14.99)	4.99(1.90–13.13)
Poor	1.54(0.71–3.34)	2.56(1.12–5.86)
**Type of ARV regimen**		
1a(d4T+3TC+NVP)	Reference	Reference
1b(d4T+3TC+EFV)	1.15(0.61–2.18)	0.29(0.12–0.69)
1c(AZT+3TC+NVP)	0.82(0.48–1.41)	0.45(0.22–0.89)
1d(AZT+3TC+EFV)	4.19(1.89–9.28)	4.47(1.81–11.04)
1e(TDF+3TC+EFV)	1.20(0.651–2.20)	1.14(0.59–2.20)
1f(TDF+3TC+NVP)	1.32(0.66–2.62)	1.18(0.57–2.43)

^@^ univaraite model,

* multivariable model

## Discussion

This study found 113 treatment failures per 11830 person years of observation among ART clients making the overall incidence rate of 9.38 (95% CI: 7.79–11.30) per 1000 person years. This incidence rate is relatively low as compared to the incidence rate reported by different studies in Ethiopia and abroad [10, 14, 15]. A study from Mozambique reported that rate of immunologic failure was 17.2 (95% CI: 12.6–22.9) per 100 person-years of follow-up [16]. Another study from Thailand showed that 4.3%, 10.7%, and 4.9% met the criteria of virological failure, immunological failure, and clinical failure, respectively. The probable justification could the difference in ART initiation criteria, nutritional factor and follow up approach between Ethiopia and those countries. A relatively similar incidence rate of treatment failure was reported from Ghana [17]. A study from Debremarkos Hospital, Northwest Ethiopia revealed that the rate of immunological failure was 8 per 100 person-years of follow up [10].

This study found that the highest rate of treatment failure was occurred between 6 months and 10 months of ART initiation. Studies agreed that most of the treatment failure are occurred during early days of the ART initiation [14, 18]. The test for trend over time showed a decrease in the rate of immunological failure with each additional year since the initial immunological success [14]. Treatment failure that occurred within the first early months of therapy might be resulted from poor adherence, poor status disclosure, severe drug toxicity and regimen changes [19]. Promoting early disclosure of ART status, counseling on the adherence of drugs, appointing clients more frequently to visit the clinic and linking ART clients with the community health workers during early phases of treatment might be important to reduce the incidence of treatment failure in the first 12 months of ART initiation. One of the factors found to be associated with treatment failure was gender. Male ART clients were more likely to experience treatment failure compared to female ART clients. A similar finding was reported by studies from two African countries which showed that men on ART were more vulnerable to virologic failure than women [20, 21]. There have been many studies which showed that mortality among male ART clients was significantly higher as compared to their female counter parts [22–30]. One of the possible reasons could be the difference in mean CD4 count between male and female during ART initiation. Furthermore studies showed that males had unhealthy behaviors like using alcohol, cigarette, Khat etc than females [31–35] which might lead them to poor drug adherence and reduce the overall treatment success. Another study revealed that intravenous drug users were at higher odds to experience treatment failure as compared to their counterparts [14].

A retrospective case control study in India identified older age as a factor for treatment failure [36]. This finding showed that increasing age is associated with increased odds of treatment failure. Similarly, it was found that those bedridden and ambulatory clients were more likely to experience treatment failure compared to working clients at their ART initiation period. In this regard, a study from Ethiopia reported supporting evidences that inability to work due to health problem is associated with treatment failure [10]. The high prevalence of mortality among ART clients with poor baseline functional performance might be due to the treatment failure as evidenced in this study [28, 37, 38]. Lower baseline CD4 count was significantly associated with higher odds of treatment failure. There are studies which showed that lower baseline CD4 count especially less than 100m^3^/dl was associated with higher odds of treatment failure [39, 40].

TB co-infection is one of the most consistent predictor for treatment failure among ART patients. This study also found that those ART clients who had TB co-infection during ART initiation were 3 fold higher to experience treatment failure as compared to their counterparts. Studies from Ethiopia and abroad reported similar finding which underline the importance of TB in treatment success [10, 36]. Not only co-infection during ART initiation but also developing TB after ART initiation was one of the significant factors associated with treatment failure. Similarly, having other opportunistic infection during ART initiation was significantly associated with treatment failure. Consistent finding was also reported from Debremarkos Hospital which revealed that having pneumonia was significantly associated with higher odds of having treatment failure [10].

This study found that poor drug adherence was associated with higher odds of having treatment failure. A consistent finding was reported from Tanzania which showed that history of poor antiretroviral therapy adherence due to exposure to drug holiday was associated with treatment failure [15]. Another study from Kenya showed that imperfect ART adherence as associated factor for treatment failure [41]. A study from Ethiopia reported that treatment interruption associated with higher odds of treatment failure [10].

In this study it was found that there is variation in the experience of treatment failure among drug regimens. Those patients who had used 1b (d4T+3TC+EFV) and 1c(AZT+3TC+NVP) had lower odds of treatment failure as compared to 1a(d4T+3TC+NVP) patients. On the other hand those patients who had used 1d(AZT+3TC+EFV) drug regimen had higher odds to experience treatment failure as compared to 1a based treatment regimen. A case control study from Kenya revealed that those ART clients who were on Zidovudine based ART regimen experienced treatment failure as compared to other regimen based treatments [41].

### Limitations of the study

This study has some limitations. The first limitation was that this study conducted based on secondary data analysis which missed key variables those should be considered. The other limitation was in this study, clinical failure and immunological failure were used for measuring treatment failure which usually underestimate the treatment failure. Though the study used clinical failure and immunological failure to determine the rate of treatment failure, we could not present the figures of treatment failure by type as immunological failure and clinical failure separately due to the fact that the treatment failure is not presented separately by type in the database system for all patients. The gold standard method for measuring treatment failure is the virological assessment however, which was not used in this study. Incomplete data was also one of the major limitations of this study.

## Conclusion

The rate of treatment failure needs attention in the Bale zone Hospitals. The highest rate of treatment failure was occurred between 6 months and 10 months of ART initiation. Factors associated with treatment failure were age of clients, male ART clients, bedridden clients, ambulatory clients, TB co-infected clients at ART initiation, clients who develop TB co-infection after ART initiation, type of drug regimen used, drug adherence, clients with low CD4 count at ART initiation, and clients who have reported other opportunistic infection. Those factors should be considered in prevention of treatment failure among ART clients. Continual follow up and focused care especially in the first 12 months of ART initiation is important to identify early treatment failure using the available resources.

## Supporting Information

S1 File Data set from which this manuscript produced. (XLSX)
